# Data on the growth of ZnO nanorods on Nylon 6 and photocatalytic activity

**DOI:** 10.1016/j.dib.2016.06.014

**Published:** 2016-06-21

**Authors:** S. Ummartyotin, B. Tangnorawich

**Affiliations:** a Advanced Functional Polymeric Materials Research Group, Faculty of Science and Technology, Thammasat University, Patumtani 12120, Thailand; b Materials Research Center, In collaboration with HORIBA Scientific and Thammasat University, Patumtani 12120, Thailand; c Department of Physics, Faculty of Science and Technology, Thammasat University, Patumtani 12120, Thailand

**Keywords:** ZnO, Rod-like, Nylon 6, Photocatalytic activity

## Abstract

ZnO was successfully synthesized by a conventional synthetic route using zinc nitrate as a source for ZnO formation. X-ray diffraction and thermogravimetric analysis revealed a crystal size of 66 nm of ZnO and a thermal stability of 500 °C. A small amount of ZnO particles was employed as the source for ZnO-rod growth on nylon 6 surfaces. Scanning electron microscope images were taken to evaluate the morphological properties of ZnO, which presented as a hexagonal needle-like shape. Preliminary evaluation of photocatalytic activity was performed through measurement of the degradation of methylene blue solution over 4 h.

**Specifications Table**

**Table:** 

Subject area	*Physics*
More specific subject area	*Characterization of the ZnO rod and photocatalytic properties*
Type of data	*Figure*
How data were acquired	*XRD, FTIR, SEM, UV–vis*
Data format	*Analyzed*
Experimental factors	*Growth of ZnO was prepared on the surface of nylon 6*
Experimental features	*ZnO was successfully synthesized from the wet chemical reaction of zinc nitrate. Thermal decomposition was employed to form ZnO particles. Rod-like ZnO particles were deposited on the surface of nylon 6. The photocatalytic activity of ZnO on the surface of nylon 6 was evaluated through the degradation of methylene blue*
Data source location	*Faculty of Science and Technology, Thammasat University, Thailand*
Data accessibility	*Data are provided in this article*

**Value of the data**

• ZnO was successfully synthesized through a wet chemical reaction of zinc nitrate.
• ZnO particles were employed as the source of ZnO rod-like particles on the surface of nylon 6.
• ZnO-coated nylon 6 can be used as an excellent material for photocatalytic degradation of methylene blue.

## 1 Data

**Fig. 1:**
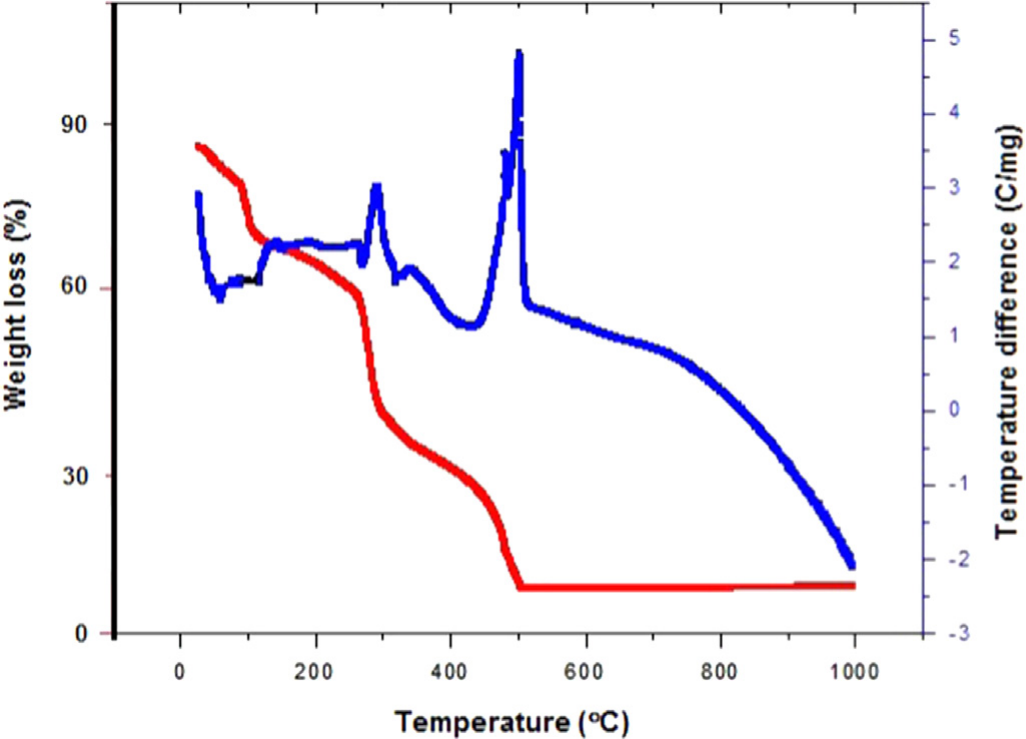
Thermogravimetric analysis of the Zn–O complex.

**Fig. 2:**
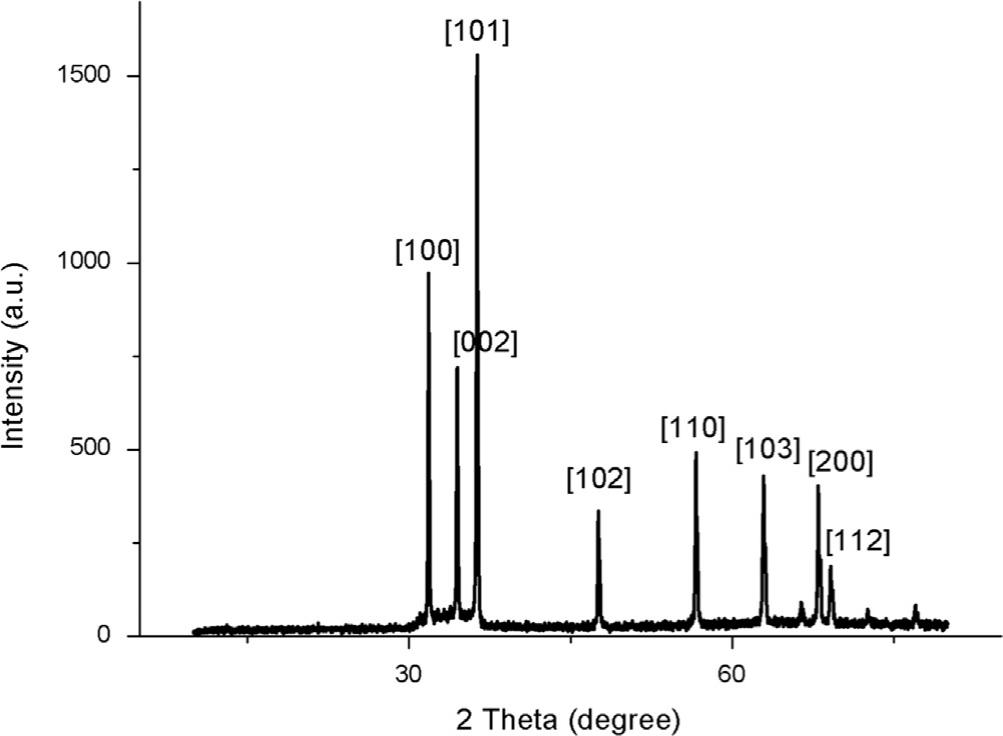
X-ray diffraction pattern of ZnO.

**Fig. 3:**
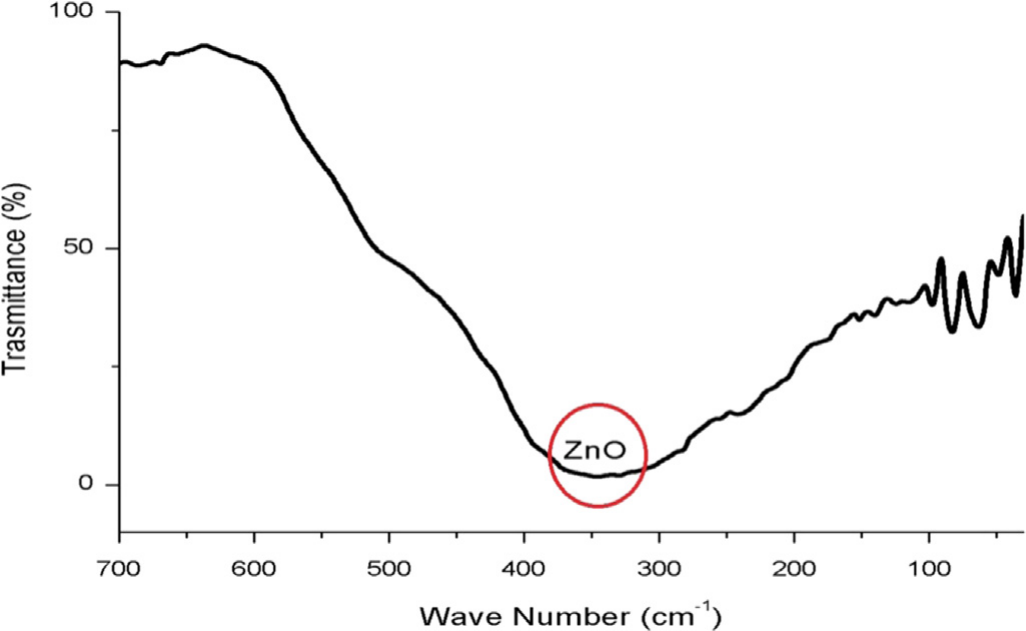
Fourier transform infrared of ZnO and Nylon 6.

**Fig. 4:**
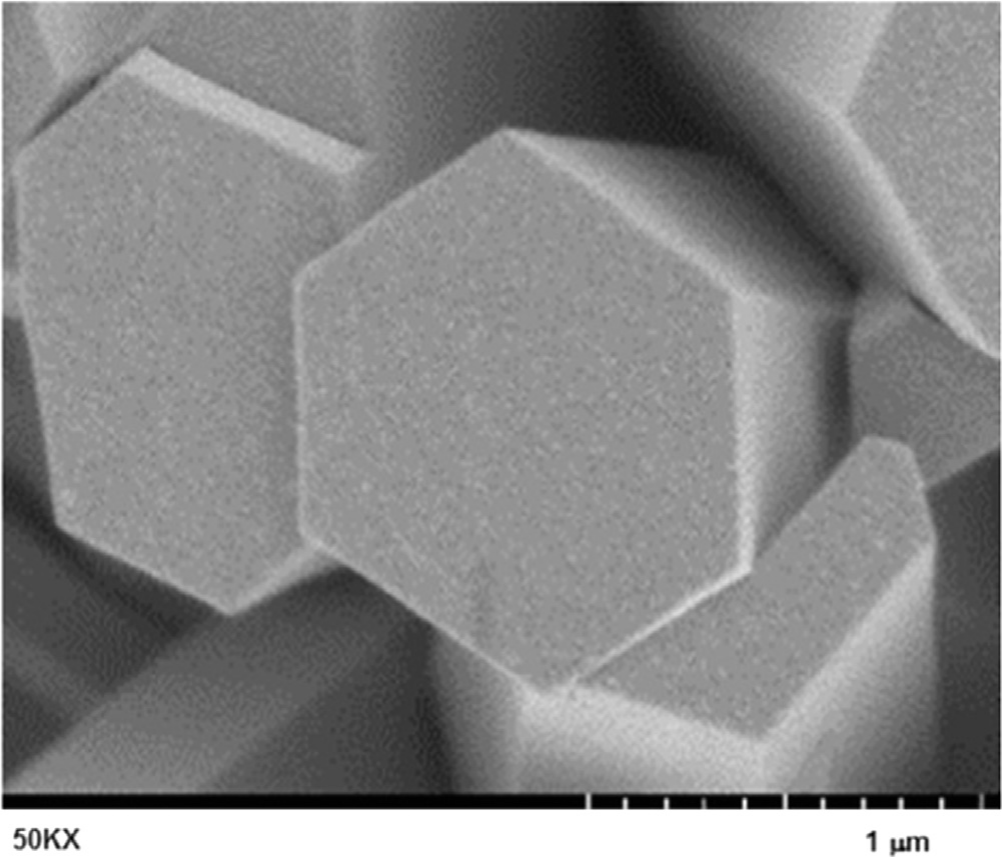
Morphological properties of ZnO growth on ZnO at a magnification of 50000X.

**Fig. 5:**
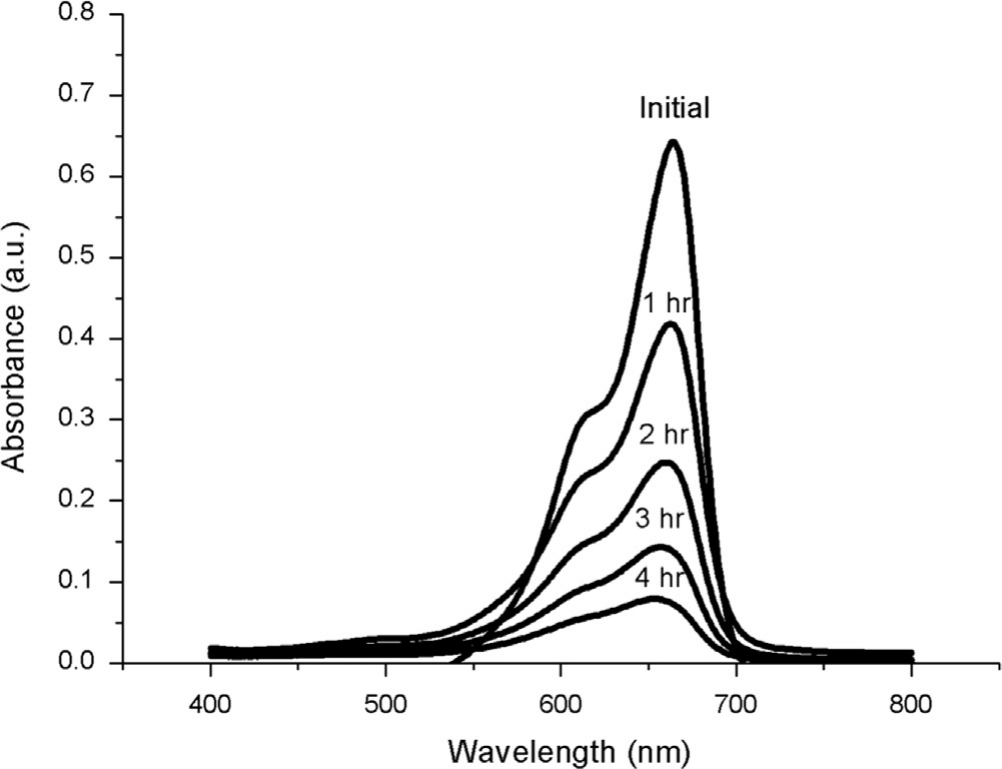
Absorption change of methylene blue in the presence of ZnO on Nylon 6 surface under sunlight irradiation.

ZnO is thermally stable over 500 °C (Fig. 1). XRD and FTIR present the structural properties and Zn–O bonding in Fig. 2, Fig. 3, respectively. Morphological properties of ZnO were investigated using SEM (Fig. 4). Preliminary evaluation of methylene blue degradation was carried out over 4 h (Fig. 5).

## 2 Experimental design, materials and methods

The TGA analysis shown in Fig. 1 demonstrates that ZnO degradation was stable at temperatures over 500 °C. However, it exhibited char and incomplete degradation. Calcination was conducted from 700 °C. The weight loss during calcination can be categorized into three temperature regions. From room temperature to 200 °C, it involved water and solvent evaporation. From 200–500 °C, it was due to organic decomposition, and from 500 °C and higher, the weight loss was stable. Formation of ZnO should be performed in this temperature region.

Following calcination at 700 °C, the structural properties of the power were investigated by X-ray diffraction. Fig. 2 shows the X-ray diffraction pattern. It was found that sample exhibited a single phase that can be identified as the hexagonal wurtzite structure of ZnO (space group P6_3_mc). This is in agreement with JCPDS no. 36-1451. No trace of other impurities was found within the detection limit of instrument [1], [2], [3], [4]. From the peak, the preferential orientation was determined using a texture coefficient (hkl). It was illustrated that the highest value was in the (101) plane for the sample, which indicated that crystal orientation is uniform in the x- and z-orientation. The crystal size was estimated by the Scherrer formula: *D*=*Kλ*/*β*cos*θ*, where *D* is crystallite size, *K* is a constant of 0.9, *λ* is the X-ray wavelength, *β* is full width at half maximum (FWHM) and *θ* is the diffraction peak [5], [6]. The (101) peak was used to estimate the crystal, and it was found to be 66 nm.

After structural characterization of ZnO powder, it was suspended in methanol solution at a concentration of 5 wt% and spin-coated on Nylon 6 substrate. Fig. 3 exhibits the FTIR pattern of ZnO coated on the Nylon 6 substrate. FTIR pattern of the sample was obtained between 500 and 4000 cm^−1^. The chemical group of Nylon 6 was observed. The broad transmission peak in the region of 3000–3500 cm^−1^ corresponded to the –OH group, indicating water absorption on the surface of Nylon 6. The peak region of 2500–3000 cm^−1^ is due to C–H stretching modes. This peak is due to the C–H bonds that compose the main structure of the Nylon 6 polymer. The peak between 1400 and 1600 cm^−1^ is due to the absorption of atmospheric CO_2_ on the surface of Nylon 6, as discussed previously [7].

To gain more efficiency on the use of ZnO film, it was tested via a hydrothermal process. Fig. 4 shows the morphological properties of as observed through scanning electron microscopy at a magnification of 50000X. The image shows the cross-sectional area of the needle-like hexagonal rod with a diameter of 20 nm at a high aspect ratio (L/D) of 10. The diameter and length of the ZnO rod were estimated to be 20 and 200 nm, respectively. In addition, it was found that the ZnO rod was uniformly epitaxially deposited on the Nylon 6 substrate, suggesting a smooth surface and similar level of surface energy before deposition.

Fig. 5 illustrates the photocatalytic activity of ZnO nanorod on Nylon 6 using methylene blue as model pollutant. The UV–vis absorption of methylene blue was reduced over time in the presence of ZnO under sunlight irradiation. Before the reaction, an absorption peak at 650 nm attributed to the absorption of n→π* transition of methylene blue can be observed, as discussed previously [8]. Furthermore, it can be observed that the methylene blue absorption dramatically decreases as the reaction time increased. According to the change of absorption intensity at 650 nm, the decolorization efficiency of methylene blue solution with the existence of ZnO was estimated to be 60%. It was important to note that the experiment should be conducted within 6 h. The photocatalytic activity of Nylon 6 can be enhanced with increased reaction time, amount of ZnO and partial transition metal ion substitution.
