# Supplementary material for: Data on the growth of ZnO nanorods on Nylon 6 and photocatalytic activity
Source: Data Brief. 2016 Jun 21;8:643–7. doi: 10.1016/j.dib.2016.06.014 (PMC4939396; doi:10.1016/j.dib.2016.06.014)
Supplement: Supplementary file 1 — Supplementary material [file mmc1.doc]

**Conflict of Interest form**

There is no conflict interest on this research work.
