# Supplementary material for: Data on the growth of ZnO nanorods on Nylon 6 and photocatalytic activity
Source: Data Brief. 2016 Jun 21;8:643–7. doi: 10.1016/j.dib.2016.06.014 (PMC4939396; doi:10.1016/j.dib.2016.06.014)
Supplement: Supplementary file 2 — Supplementary material [file mmc2.pdf]

Language Editing Services

Registered Office:
Elsevier Ltd
The Boulevard, Langford Lane,
Kidlington, OX5 1GB, UK.
Registration No. 331566771

To whom it may concern

The paper "Data on the Growth of ZnO nanorod on Nylon 6 and photocatalytic activity" by Sarute Ummartyotin was edited by Elsevier Language Editing Services.

Kind regards,

Biji Mathilakath
Elsevier Webshop Support

(This is a computer generated advice and does not require any signature)
